# Dual-track drive for precision education: developing a targeted teaching model in the standardized training of lymphoma subspecialty physicians through the integration of problem-based learning and case-based learning

**DOI:** 10.1186/s12909-025-08200-9

**Published:** 2025-11-12

**Authors:** Pengjun Liao, Sichu Liu, Chengwei Luo, Xiaojuan Wei

**Affiliations:** 1 Department of Hematology, Guangdong Provincial People’s Hospital (Guangdong Academy of Medical Sciences), Southern Medical University, Guangzhou, 510080 China; 2 Department of Lymphoma, Guangdong Provincial People’s Hospital (Guangdong Academy of Medical Sciences), Southern Medical University, Guangzhou, 510080 China

**Keywords:** Standardized training of lymphoma subspecialty physicians, Problem-based learning, Case-based learning, Teaching model

## Abstract

In the training of lymphoma subspecialty physicians, confronted with the challenges of rapid knowledge updates and complex diagnosis and treatment decisions, this paper proposes a dual-track teaching model that integrates problem-based learning (PBL) and case-based learning (CBL). This model prompts trainees to actively explore knowledge through well-designed clinical problem chains and simulates the clinical decision-making process using selected real cases, aiming to enhance trainees’ independent diagnosis and treatment capabilities, critical thinking, and evidence-based decision-making skills. The teaching implementation emphasizes teacher guidance, group collaboration, and multidimensional ability assessment. This model can effectively stimulate learning motivation and promote the integration of clinical thinking and interdisciplinary knowledge. In the future, it is necessary to continuously optimize teacher capabilities, update case banks, and explore the application of new technologies.

## Introduction

Medical education is undergoing a fundamental shift from a primary focus on knowledge transmission to an emphasis on cultivating competencies such as critical thinking, self-directed learning, and clinical reasoning [1]. This shift is particularly critical in highly dynamic and complex subspecialties like lymphoma. Training lymphoma specialist residents faces significant challenges: the rapid evolution of WHO classification standards and the complexity of pathological diagnoses [2]; the continuous emergence of novel therapies. leading to diverse and nuanced treatment decisions [3, 4]; and the persistent need to disseminate standardized diagnostic and treatment approaches effectively. Traditional teaching methods, predominantly characterized by large-group lectures and passive case demonstrations, are insufficient to address these challenges. They often fail to replicate the ambiguity and pressure of real-world clinical scenarios, provide limited opportunity for active trainee participation, and consequently prove ineffective in systematically cultivating the essential skills of critical analysis and evidence-based decision-making [5].

Problem-based learning (PBL) and case-based learning (CBL) are two established learner-centered methods in medical education reform. PBL uses complex, real-world problems as a trigger for *group-based* inquiry, through which trainees *independently* identify learning needs, seek out evidence, and construct knowledge, thereby fostering self-directed learning skills [6–9]. Conversely, CBL uses carefully crafted real cases to provide a structured environment for trainees to practice applying knowledge and simulating standardized diagnosis and treatment processes [10, 11]. The combination of PBL (driving knowledge acquisition) and CBL (providing a context for application) is highly suitable for lymphoma, a specialty defined by fine pathological typing, highly individualized treatment, and reliance on multidisciplinary team (MDT) collaboration [12–14]. 

This paper presents a detailed conceptual framework and implementation plan for the PBL + CBL dual-track model, laying the groundwork for its future implementation and empirical evaluation. The rapidly evolving and highly complex nature of lymphoma—requiring integrative diagnostic capabilities, evidence-based decision-making, and effective MDT collaboration—underscores the limitations of traditional lecture-based instruction, which often fails to simulate real-world clinical pressures or cultivate critical thinking skills. By detailing the curriculum design, implementation process, and evaluation framework of this model, this study aims to provide a practical and scalable educational approach to bridge existing gaps in training and develop lymphoma specialists who are proficient in molecular diagnostics, current evidence application, and collaborative practice.

## Overview of the PBL and CBL teaching models

### PBL teaching model

Problem-based learning (PBL) is a student-centered pedagogical approach that uses complex, real-world problems as the starting point for learning, contrasting with traditional didactic lectures [15]. In PBL, learning is driven by the process of group-based inquiry, where students, facilitated by a tutor, identify their learning needs, actively research the problem, and collaboratively construct knowledge [9]. The primary strength of PBL lies not merely in knowledge acquisition but in cultivating core competencies essential for modern physicians, including self-directed learning, critical thinking, and problem-solving skills [6, 7].

### CBL teaching model

Case-based learning (CBL) is another learner-centered method that uses carefully crafted, authentic clinical cases to simulate real-life practice [10]. While PBL uses a problem to ‘drive the acquisition’ of new knowledge, CBL uses a case as a context for trainees to ‘apply’ existing and newly acquired knowledge to solve clinical problems, thereby bridging the gap between theory and practice [11].

In CBL classes, teachers provide case materials, including medical history, symptoms, test results, imaging reports, and treatment effects. Students need to understand the cases, link textbook knowledge, analyze and reason, make decisions, and summarize and reflect. The CBL teaching method integrates theory with practice, cultivates clinical thinking, and improves decision-making ability within a simulated clinical context [16].

Compared with PBL, CBL focuses more on applying knowledge and cultivating specific clinical skills, whereas PBL focuses more on driving students to actively explore new knowledge [17]. The two are often used in combination to achieve better teaching effects [18].

## Application of PBL combined with the CBL teaching model in the standardized training of residents in the lymphoma subspecialty of hematology

Having outlined the core principles of PBL and CBL, the following sections detail the rationale and practical implementation of integrating these two methods specifically within the context of lymphoma subspecialty training, where their complementary strengths are particularly salient.

### Application background: teaching needs to cope with the complexity of lymphoma diagnosis and treatment

Lymphoma knowledge is updated rapidly, and its diagnosis, treatment, and subtyping are highly complex. These characteristics create major challenges for physician training: accurate diagnosis requires integrating multiple diagnostic tests; individualized treatment demands balancing novel therapies with patient-specific factors; and residency training must strengthen skills in standardized diagnosis and evidence-based decision-making [2, 4, 19]. Traditional theoretical teaching and case demonstrations are unable to meet these needs because of the lack of real decision-making pressure and the active participation of trainees.

To address this, we adopt a dual-track model that combines PBL (problem-based learning) and CBL (case-based learning). PBL drives trainees to actively study knowledge through complex problems; CBL provides a practice field with real cases, aiming to clearly improve abilities. This requires facilitators to possess *more advanced instructional skills*, including curriculum design, guided facilitation, and the ability to provide timely, effective feedback.

### Application process: the key lies in carefully building a closed loop of teaching and learning

Effective integration of PBL and CBL in lymphoma specialty training requires a carefully designed process: stimulating learning through structured problems, using authentic cases as practice fields, and progressively enhancing trainees’ abilities. This requires higher qualities from teachers, including curriculum design, guiding discussions, and timely and effective feedback.

#### Teaching design: targeting goals and creating a “Problem + Case” core

Teaching design is crucial for the successful implementation of the entire PBL + CBL model. The most important step in this process is to carefully design “problems” and “cases” that can truly stimulate learning motivation and align perfectly with the training goals of the lymphoma specialty—this serves as the engine of the entire teaching process: ① Accurately anchor goals: clarify the core knowledge (typing, staging, treatment), skills (interpreting reports, evidence-based decision-making, communication), and attitudes (evidence-based thinking, individualized concepts) that trainees need to master in a specific unit (such as DLBCL diagnosis and treatment). ② Select real cases: cases need to be typical (focusing on reflecting diagnosis and treatment challenges), real and complete (providing complete clinical data), and hierarchical (able to trigger in-depth discussions), which form the basis for learning and problems. ③ Designing progressive problem chains: Design structured problems around cases to guide trainees to think according to the clinical path (collecting information ->diagnosis ->evaluation ->decision-making ->communication). Problems need to target core knowledge and skills, simulate real decision-making dilemmas, and stimulate critical discussions and evidence evaluation.

##### Explicit example: a sample case and problem chain for DLBCL

*Case Presentation: A 45-year-old woman presents with a rapidly growing neck mass. Excisional biopsy pathology report is provided*,* indicating Diffuse Large B-Cell Lymphoma (DLBCL). Initial staging PET-CT shows widespread nodal and extranodal involvement. Laboratory tests include elevated LDH and performance status (ECOG 1).*

*Structured Problem Chain*:

1. *Diagnosis & Classification: Based on the pathology report (e.g.*,* IHC: CD20+*,* CD10-*,* BCL6+*,* MUM1+)*,* what is the cell-of-origin subtype according to the Hans algorithm? What additional molecular studies might be crucial for risk stratification and why?*
2. *Staging & Prognosis: Using the provided data*,* what is the Ann Arbor stage and IPI score? What is the prognostic implication?*
3. *Treatment Decision-Making: What is the standard first-line therapy? Are there factors in this case that might justify considering an intensified regimen over R-CHOP? Discuss the evidence.*
4. *MDT Simulation & Communication: If you were to present this case in an MDT meeting*,* what key information would you highlight for the pathologist and radiation oncologist? How would you explain the treatment plan to the patient?*

#### This example illustrates how a real case is structured with progressive problems to guide learners through the clinical pathway, stimulating critical thinking and evidence application

##### Resource preparation and grouping strategy

1. *Building learning scaffolds*: Prepare or indicate key learning resources for trainees, such as the latest version of the NCCN/CSCO lymphoma diagnosis and treatment guidelines, core textbook chapters, key review literature, and authoritative databases (such as UpToDate). Avoid “searching for a needle in a haystack” while refraining from providing “standard answers”.
2. *Optimizing group composition*: Heterogeneous grouping is conducted on the basis of trainees’ knowledge background, ability characteristics, and personalities (usually 4–6 people per group) to ensure that members in the group can complement and assist each other. Clarify the division of tasks in the group (such as information retrieval, information sorting, and report preparation) and set clear group output goals (such as diagnosis reports, treatment plans, and dispute analysis reports).

#### Teaching implementation: inquiry and collaboration under dynamic guidance

Teaching implementation is a dynamic process that transforms a carefully designed blueprint into a real learning experience, with the tutor’s role shifting from “lecturer” to “guide” and “catalyst”.

##### Case introduction and problem initiation

Tutors present the core background information of the case concisely and focusedly (such as “A 65-year-old male presents with progressive lymphadenopathy, and the initial pathology suggests large B-cell lymphoma”), quickly bringing trainees into the clinical context. The preset core guiding problem chain (e.g., “First, we need to clarify the diagnosis and prognosis; second, formulate an individualized treatment plan; finally, consider possible complications and follow-up plans”), and clarify the core tasks and exploration directions of this learning. Information overload or vague problems should be avoided.

##### Group inquiry and tutor guidance

- ① Independent inquiry period: Trainees, according to group tasks, use provided resources and self-retrieval to conduct in-depth information collection, sorting, analysis, and preliminary discussions around assigned problems. This stage emphasizes the initiative and autonomy of trainees.
- ② Dynamic guidance (Facilitation): Tutors patrol among groups and play key roles.
  - Observer: Close monitoring of the discussion dynamics, participation, obstacles encountered, and thinking trends of each group.
  - Questioner: Timely put forward focused and heuristic questions (socratic questioning), such as “What is the evidence level of the treatment plan proposed by your group?” “In this pathological result, CD10 is negative, BCL6 is positive, and MUM1 is positive. In combination with morphology, which type of germinal center/nongerminal center type is more likely to be involved. What is the basis?” “For this patient with a high-risk IPI score, is R-CHOP alone sufficient? What are the intensification strategies?” Guide trainees to think in depth, question assumptions, and connect theories.
  - Resource guide: When trainees are stuck or deviate from the direction, prompt key clues or suggest consulting specific resources (such as a certain chapter of the guidelines or key research).
  - Process manager: Control the discussion time to ensure the effective progress of each link.
  - Catalyst for explicit thinking: Encouraging trainees to clearly express their reasoning process and transform “tacit thinking” into “explicit discussion”.

##### Report exchange and In-depth interaction

1. *Structured report*: Each group selects a representative to clearly present the inquiry results and core conclusions, including diagnosis, staging, treatment plan selection, and key reasoning basis. Encourage the use of PPTs or blackboard writing for assistance.
2. *Focused questioning and debate*: This is a key link to deepen learning. Tutors guide questioning and challenging conclusions to enhance the understanding of evidence and decision-making complexity. Encourage constructive debates and exchanges between different viewpoints.
3. *Tutor summary, guidance, and improvement*: After the trainees’ discussion, tutors summarize the core content, clarify consensus, and correct deviations. In combination with cases, the core requirements of standardized diagnosis and treatment of lymphoma, such as the basis for diagnosis, staging, and treatment selection, should be emphasized. Summarize the ideas and decision-making principles for patient care and extract the “know-how”. Mention new research progress or clinical controversies to stimulate trainees’ interest.

##### Reflection and integration

Guide trainees to conduct individual and group reflections: review the entire learning process, think about their gains and deficiencies in knowledge acquisition, thinking methods, and teamwork. To encourage trainees to integrate the knowledge and skills obtained in this case study into their existing cognitive framework, this can be facilitated through structured prompts (e.g., “What was the most challenging part of the case?“) and by requiring trainees to submit a concise reflective summary.

#### Teaching effect evaluation: a proposed framework for assessing model effectiveness

The evaluation of the PBL combined with the CBL teaching model should move beyond traditional knowledge-based examinations to a comprehensive, multi-method assessment framework. This framework is designed to measure the development of core competencies throughout the learning process and upon its completion. The proposed evaluation strategy combines formative and summative assessments, alongside rigorous program evaluation, as outlined below and summarized in Table 1.

**Table 1:** Proposed evaluation framework for the PBL + CBL Dual-Track model

Assessment Type	Method	Primary Competencies Measured	Timing
Formative	Discussion Observation Rubric	Participation, Critical Thinking, Teamwork	Ongoing
	Phased Report Feedback	Analytical & Communication Skills	After each case
	Reflective Journal	Metacognition, Integration of Learning	Ongoing
Summative	Case-based Written Exam	Knowledge Application, Decision-making	End of module
	OSCE	Clinical Skills, Communication, Professionalism	End of module
	Skill Operation Assessment	Technical Proficiency	End of module
Program Evaluation	Satisfaction Survey	Learner Experience, Perceived Value	Post-course
	In-depth Interviews	Qualitative Feedback, In-depth Insights	Post-course
	Long-term Outcome Tracking	Real-world Clinical Performance	Post-graduation

##### Formative assessment: tracking competency development

Formative assessment is integrated throughout the learning process to provide continuous feedback and guide improvement.

1. *Group Discussion Observation Rubric*: Tutors use a structured rubric to record and evaluate each trainee’s performance based on predefined dimensions, such as active participation, quality of inquiry, critical thinking, teamwork, and communication skills during each session.
2. *Phased Report Feedback*: Immediate and specific feedback is provided on the content and presentation of each group’s report, focusing on logical reasoning, accuracy, depth of analysis, and clarity of expression.
3. *Reflective Journal*: Trainees maintain a learning journal to document their thought processes, challenges encountered, insights gained, and self-assessed growth in knowledge and clinical reasoning skills. This encourages metacognition and integration of learning.

##### Summative assessment: measuring learning outcomes

Summative assessment evaluates the overall achievement of learning objectives at the end of a module or course.

1. *Case-based Written Examination*: Examinations feature complex, unfolding lymphoma cases designed to assess trainees’ mastery of standardized diagnostic and treatment knowledge, as well as their clinical decision-making logic.
2. *Objective Structured Clinical Examination (OSCE - lymphoma-specific stations)*: Stations are designed to simulate key lymphoma care scenarios (e.g., breaking bad news, obtaining informed consent for chemotherapy, discussing treatment options in an MDT setting). This assesses clinical communication, information integration, decision-making ability, and professionalism.
3. *Skill Operation Assessment*: Trainees’ proficiency in core procedures relevant to lymphoma care, such as bone marrow aspiration and biopsy or lumbar puncture, is assessed for technical standardization, indication comprehension, and management of complications.

##### Program evaluation: assessing the model itself

To establish the educational value of this dual-track model (a key aspect of scholarship), a systematic program evaluation will be conducted.

1. *Structured Trainee Satisfaction and Perception Survey*: A validated questionnaire will be administered post-course to understand trainees’ perceptions of the model’s effectiveness, engagement level, and its impact on their confidence and clinical reasoning abilities. This will provide quantitative data on the learner experience.
2. *Small-scale In-depth Interviews*: A subset of trainees and facilitators will be invited to participate in semi-structured interviews to gain rich, qualitative insights into the learning experience, perceived strengths and weaknesses of the model, and suggestions for improvement.
3. *Long-term Outcome Tracking (Prospective)*: A crucial component of future scholarship will be to track graduates’ performance in clinical practice, monitoring indicators such as diagnostic accuracy, adherence to treatment guidelines, and MDT collaboration skills, thereby assessing the long-term translational impact of the training.

### Advantages of the PBL + CBL combination in lymphoma training

The advantages discussed below for the PBL + CBL model are theoretically derived from established educational principles and the specific needs of lymphoma training. Empirical validation through future controlled implementations is planned to substantiate these anticipated benefits.

#### Stimulating trainees’ learning enthusiasm

In the rapidly evolving field of lymphoma, the PBL + CBL model is designed to stimulate trainees’ intrinsic motivation by introducing unresolved clinical problems (for example, “Why is a young patient unresponsive to the R-CHOP regimen?“). Trainees need to analyze complex pathological reports, evaluate and adjust chemotherapy doses, and apply theoretical knowledge to practical clinical problems. This practice-oriented learning taskapproach is expected to prompt trainees to actively consult literature and find evidence to expound their views in group discussions.

#### Cultivating trainees’ judgment

There is no fixed model for lymphoma treatment. In the PBL session, trainees need to independently find key studies and analyze relevant data. Facing in-depth questions from tutors (such as “Which guideline recommendation is the treatment plan on which? “), trainees must examine whether their evidence is sufficient. Through this process of independent evidence retrieval and critical appraisal in response to facilitator questioning, the model aims to train standardized training doctors to think independently and establish robust evaluation logic (for example, weighing the potential benefits and risks of intensive chemotherapy), thereby deeply understanding the value of evidence-based medicine.

#### Promotion of team collaboration

The cases designed by the CBL simulate the complete diagnosis and treatment process of patients from initial diagnosis to follow-up. Trainees need to experience diagnosis, staging, treatment decision-making, and follow-up, similar to attending physicians (for example, mantle cell lymphoma [MCL] patients). Trainees personally experience multidisciplinary collaboration (such as communication with pathology and radiotherapy departments), understanding the importance of teamwork for standardized diagnosis and treatment and individualized treatment (precision medicine). Simulating MDT consultations enables trainees to realize that multidisciplinary cooperation is indispensable.

#### Integrating the knowledge of various disciplines

The diagnosis and treatment of lymphoma require multidisciplinary knowledge. For example, when dealing with primary central nervous system lymphoma (PCNSL) cases, trainees need to integrate imaging (interpreting brain MRI), pharmacology (calculating methotrexate dose while considering blood‒brain barrier penetration), and neurology (preventing and handling neurotoxicity) knowledge to formulate treatment plans. To face complex challenges, trainees must integrate knowledge from different disciplines to form feasible plans. This training helps overcome the limitations of single-specialty thinking and cultivates trainees’ ability to comprehensively consider curative effects and patients’ quality of life when formulating treatment plans, which is the core ability of lymphoma specialists.

## Realistic challenges: difficulties in PBL + CBL teaching in a lymphoma subspecialty

While the integrated PBL + CBL model presents a theoretically robust solution to the training challenges outlined in the introduction, its practical implementation faces several foreseeable hurdles. Acknowledging and planning for these challenges is crucial for successful adoption.

### Ideal is Full, reality has reefs

#### Designing problems is like walking a tightrope

Designing effective problem chains requires balancing three conflicting requirements: problems that are too shallow are boring, whereas those that are too deep are difficult to understand; cases that are too comprehensive lack focus, whereas those that are too focused lose authenticity; and controversial problems can stimulate speculation but may confuse beginners. Standardized trained doctors need to find a balance among these challenges. For instance, a problem asking “What is the definition of DLBCL?” is too simplistic, while “Design a novel CAR-T construct for a patient with triple-hit lymphoma resistant to standard therapy” may be overwhelming for a beginner. The key lies in finding the appropriate level of challenge, for example: ‘Compare the evidence for R-CHOP vs. DA-EPOCH-R in a young patient with a high-risk IPI score.

#### Selecting cases is more difficult than consulting in difficult pathologies

Case selection needs to avoid being as boring as the “model plays” or as intimidating as “overly difficult questions”; knowledge is updated rapidly, and old cases may become outdated; real case teaching needs to protect privacy without losing authenticity. A common but straightforward case of limited-stage Hodgkin lymphoma might not engage learners effectively, while an exceedingly rare and complicated case of hemophagocytic lymphohistiocytosis associated with lymphoma might divert focus from core principles. Selecting a case of primary refractory DLBCL, which is complex yet representative of a critical clinical challenge, exemplifies the difficulty in case curation.

#### Group discussions reflect “Department Ecology”

In discussions, “molecular geeks” may dominate, while “hands-on enthusiasts” remain silent; some members may not participate due to high pressure or fatigue; “microphone hogs” often appear in the report session; and the ideas of introverts are often overlooked.

### Proposed strategies to address challenges

#### Strengthening teacher capabilities

① Organize “mentoring for new teachers”: Experienced teachers (such as pathology experts and heads of new drug trials) guide new teachers to interpret complex cases and discuss cutting-edge controversies (such as the selection of regimens for refractory cases). ② Establishing a dynamic case bank: Regually collect real difficult cases (diagnostic difficulties, treatment problems, ethical dilemmas) and classify them by difficulty (basic standardized type, controversial speculative type, multidisciplinary integrated type). Crucially, beyond content expertise, specific training in facilitation skills is essential for teachers. This includes training on how to ask open-ended questions, manage group dynamics, and provide feedback without lecturing.

#### Optimizing grouping strategies

① Simulating MDT grouping: Assign roles according to trainees’ strengths (such as good at morphology - pathological analysis; good at regimens - treatment decision-making; strong at retrieval - evidence-based support; good at communication - doctor‒patient/MDT statement). ② Forcing role rotation: Require trainees to take on different roles in different cases (such as “pathological analyst” becoming a “communicator” next time) to overcome the limitations of abilities.

#### Improving incentive mechanisms

① Process incentives: immediately recognize classroom contributions (such as putting forward key differential points); use the “think-pair-share” method to encourage introverted trainees to participate. ② Result incentives: issue the “Lymphoma Subspecialty Competence Certification” with ability assessment upon graduation, reflecting the core competitiveness in employment.

## Conclusions and prospects

This paper proposed a dual-track PBL + CBL teaching model to directly address the significant challenges in lymphoma subspecialty training identified in the introduction—namely, the rapid knowledge updates, complex decision-making, and necessity for MDT collaboration that are inadequately met by traditional pedagogical approaches.

Future efforts should focus on several specific areas to translate this proposal into practice and scholarship:

1. Development of Robust Assessment Tools: Creating and validating specific instruments to measure the model’s impact on core competencies like clinical reasoning and MDT collaboration skills.
2. Longitudinal Outcome Evaluation: Implementing the model and conducting studies to track its long-term effect on graduates’ clinical performance.
3. Integration of Educational Technology: Exploring the application of advanced technologies such as virtual simulation platforms to create immersive lymphoma cases.
4. Faculty Development Programs: Systematically training facilitators to ensure high-quality guidance in PBL/CBL pedagogy.

When successfully implemented, this integrated model shifts medical education from passive knowledge transmission to active skill development, aiming to cultivate a new generation of highly competent lymphoma specialists.

## Data Availability

No datasets were generated or analysed during the current study.

## Abbreviations

PBL: Problem-Based Learning
CBL: Case-Based Learning
CAR-T: Chimeric Antigen Receptor T-cell therapy
MDT: Multidisciplinary team
DLBCL: Diffuse large B-cell lymphoma
OSCE: Objective Structured Clinical Examination
MCL: Mantle cell lymphoma
PCNSL: Primary central nervous system lymphoma
